# How the Built Environment Shapes Children’s Microbiome: A Systematic Review

**DOI:** 10.3390/microorganisms13040950

**Published:** 2025-04-20

**Authors:** Rozalynne Samira, Muntazar Monsur, Nazia Afrin Trina

**Affiliations:** 1 Department of Plant and Soil Science, Institute of Genomics for Crop Abiotic Tolerance (IGCAST), Texas Tech University, 1006 Canton Ave, Lubbock, TX 79409, USA; 2 Department of Landscape Architecture (DoLA), Davis College of Agricultural Sciences and Natural, Texas Tech University, 2904 15th St., Lubbock, TX 79409, USA; ntrina@ttu.edu

**Keywords:** built environment, design, children, microbiome, metabolites, health and well-being

## Abstract

This systematic review aims to synthesize key empirical findings to understand how various elements of the built environment influence the microbiome concerning children’s health and well-being. A comprehensive literature search was conducted across multiple databases, focusing on studies that examined the relationship between built environment factors and the microbiome aspects of childhood. A total of 42 studies were included in the final systematic review. We analyzed these studies from a range of different lenses, starting with basic research questions and variables to types of built environments, age groups of children, sampling strategy, bioinformatics, and the biological methods utilized. This review highlights a growing emphasis on children’s exposure to nature within built environments and its potential to beneficially alter the microbiome, with 38% of studies addressing this link. It also identifies a significant research gap in connecting built environment design features (landscape and/or architectural) to microbiome outcomes and associated health, behavioral, and mental health impacts on children. The findings indicate that interventions aimed at improving the built environment quality via design could foster healthier microbiomes in children’s environments. This review underscores the need for interdisciplinary research and policy initiatives that integrate microbiome science with built environment design to promote children’s health and well-being.

## 1. Introduction

Bacterial communities play a significant role in the health and well-being of children [1], and it is well-established that the surrounding built environments influence the microbial communities children are exposed to [2]. A child’s built environment (BE) may include (but is not limited to) homes, gardens, neighborhoods, schools, preschools, childcare centers, and public spaces (e.g., parks, plazas, playgrounds, zoos, museums, libraries, botanical gardens, etc.). How these diverse settings impact children’s microbiomes over time at different age levels is fascinating, complex, and multifaceted. A comprehensive understanding of this process is critically important for several reasons.

The human microbiome, a dynamic ecosystem of microorganisms including bacteria, viruses, fungi, and archaea, inhabiting various parts of the human body, particularly the skin, gut, and respiratory tract, plays a significant role in fundamental life processes and overall well-being. It is influenced by multiple factors such as genetics, diet, lifestyle, and environmental exposures [3]. In children, whose immune systems and microbiomes are still developing, the built environment is a significant contributor to microbial exposure [4]. BE–microbiome interactions start early in life and the first 1000 days (the period from conception to 2 years of age) represent the most critical window of human microbiota in healthy growth and development [5]. Childhood is a unique time in life when physical interaction with the built and surrounding environments starts or increases. Therefore, children’s exposure to microbiota, influenced by the BE, differs from that of adults and necessitates a deeper understanding of the mechanisms underlying this complex relationship. Understanding the role of BE in children’s microbiome may advance our understanding of several microbiome-induced health factors, including children’s immune development [6], protection from asthma [7], skin disease [8], allergic diseases [9], prevention from childhood cancers [10], obesity and cardiometabolic diseases [11], etc. Many adult health risk factors, including chronic cardiometabolic and heart diseases, type 2 diabetes, low immunity, asthma, orthopedic complications, psychiatric disease, and cancer risks, originate in childhood [12]. Susceptibility to both childhood obesity and asthma is intimately linked to children’s exposure to bacterial communities [13], which is intricately influenced by the nature and design of the BE that children inhabit and interact with. Approximately 32% of US children ages 2–19 are overweight or obese, which highlights a major public health problem likely to continue for many years [14]. A seminal study by Gunnell and colleagues [15] examining the relation between BMI measured in childhood and adult mortality showed that cardiovascular mortality in adults was associated with higher childhood BMIs (n = 1156 males + 1234 females). Childhood obesity also predicts the long-term risk of adult diabetes [16], and the effect is found to be independent of adult obesity [17]. In the US, the total estimated cost of diabetes alone in 2017 was USD 327 billion, including USD 237 billion in direct medical costs and USD 90 billion in reduced productivity (American Diabetes Association) [18]. Similarly, pediatric asthma imposes a substantial economic burden on the US healthcare system through increased healthcare utilization and costs [19]. The estimated combined direct and indirect cost of pediatric and adult asthma populations was as high as USD 56 billion in 2007 [20]. The data depicting the magnitude of the issues show how important it is to investigate childhood BE and its potential impact on shaping the microbiome.

This scoping review aims to synthesize current knowledge on the relationship between the BE and children’s microbiomes and identify key factors that contribute to microbiome diversity and composition. The review also summarizes findings on how BE-influenced microbiome variations are connected to different aspects of children’s health and well-being.

## 2. Methods

We started the review process by first defining the overarching premise of our question and acknowledging the complex three-way relationships between the BE, the microbiome, and children’s health and well-being. Our goal was to present a holistic review summarizing the full extent of this three-way relationship for children of all ages. Also, we aimed at connecting this review to the fields of BE design (architecture, landscape architecture, interior, and urban design, etc.) so that design professionals and researchers can extract useful information from the summary presented in the paper. We could not identify any existing systematic or scoping review that met these specific goals. We included five review papers [1,21,22,23,24] on closely related topics that highlighted the gaps we attempted to address.

Robinson and Barrable [23] presented design considerations for early childhood education settings based on the latest scientific evidence of healthy interactions between humans and environmental microbiota. The authors synthesized review findings to detailed architectural and landscape design solutions for educational settings to promote reciprocal relationships between children and their natural environments. However, this paper solely focused on the design aspects of early childhood environments, and the microbiome–BE relationships the authors reviewed were not limited to children’s BEs. Chong-Neto and colleagues [1] aimed to verify indoor and outdoor pollution, host and environmental microbiomes, and the impact on the health of the pediatric population. Based on their search criteria (keywords), their non-systematic review predominantly focused on the ‘pollution’ aspects of the environment, leaving many other potential BE variables unaddressed. Similarly, Schater and colleagues [21] focused only on soil pollution, contamination, and soil microbiome, and how, consequently, children become exposed to toxicants. Sbihi and colleagues [24] provided one of the most comprehensive reviews, including diverse aspects of BE, e.g., indoor dampness, mold, dust, outdoor soil, greenness, air, intakes of antibiotics, interactions with pets, etc. However, the review focused only on early-life environmental exposure and how such exposure shapes the gut microbiome and influences the development of allergic disease, leaving other potential dependent/outcome variables and age groups unaddressed in the review. Similarly, Mutius [22] focused on the microbial environment and its influence on asthma prevention in early life. Many other plausible physical, mental well-being, and behavioral aspects in other age groups of children were not addressed in the review.

### 2.1. Defining Childhood BE and Its Factors Influencing Microbiome

Our quest to define children’s BE started with finding a holistic definition of the BE in general. BE is an overarching term referring to “all human-made spaces in which people live, work, and recreate on a day-to-day basis” [25]. The BE encompasses places and spaces created or modified by people, including buildings, parks, and transportation systems [26]. These definitions have gradually expanded, and public health sciences have included BE quality aspects, e.g., walkability, bikeability, exposure and interaction with nature, etc., into the definition of BE. The fields of design (e.g., architecture, landscape architecture, interior design, etc.) see BE as mostly the ‘built’ aspects of the environment. There are detailed ‘built’ considerations that can influence the microbiome. For instance, design strategies that incorporate natural ventilation can facilitate the movement of outdoor microbes into indoor spaces, potentially enhancing microbial diversity compared to mechanically ventilated environments [27].

On the other hand, medical and biological sciences and environmental health research consider many behavioral and temporal aspects of the environment as part of the BE, e.g., consumption of antibiotics, breastfeeding habits, presence of pets, etc., which are significant contributors to children’s microbiome. Human activities within these spaces also play a crucial role. Children’s interactions with the environment through activities such as playing and moving around can influence microbial diversity. Moreover, the presence of other occupants, including parents, caregivers, other children, etc., contributes significantly to the microbial content of indoor environments, as human occupants are major sources of microorganisms [28].

On broader aspects of the BE, geographical location and climatic conditions of the surrounding environment affect the diversity and composition of outdoor microbes that enter indoor spaces, which then interact with human-associated microbiomes from occupants [28]. The presence and abundance of natural elements, e.g., trees, lawns, gardens, etc., (outdoor environment) and indoor plants and other greenery, green roofs, etc., (indoor environment) are significant BE contributors to children’s microbiomes. Therefore, the locations of children’s habitats (city vs. rural, farmland vs. non-farmland, etc.) also act as important BE aspects with the potential to alter children’s exposure to microbiota.

**Figure 1:**
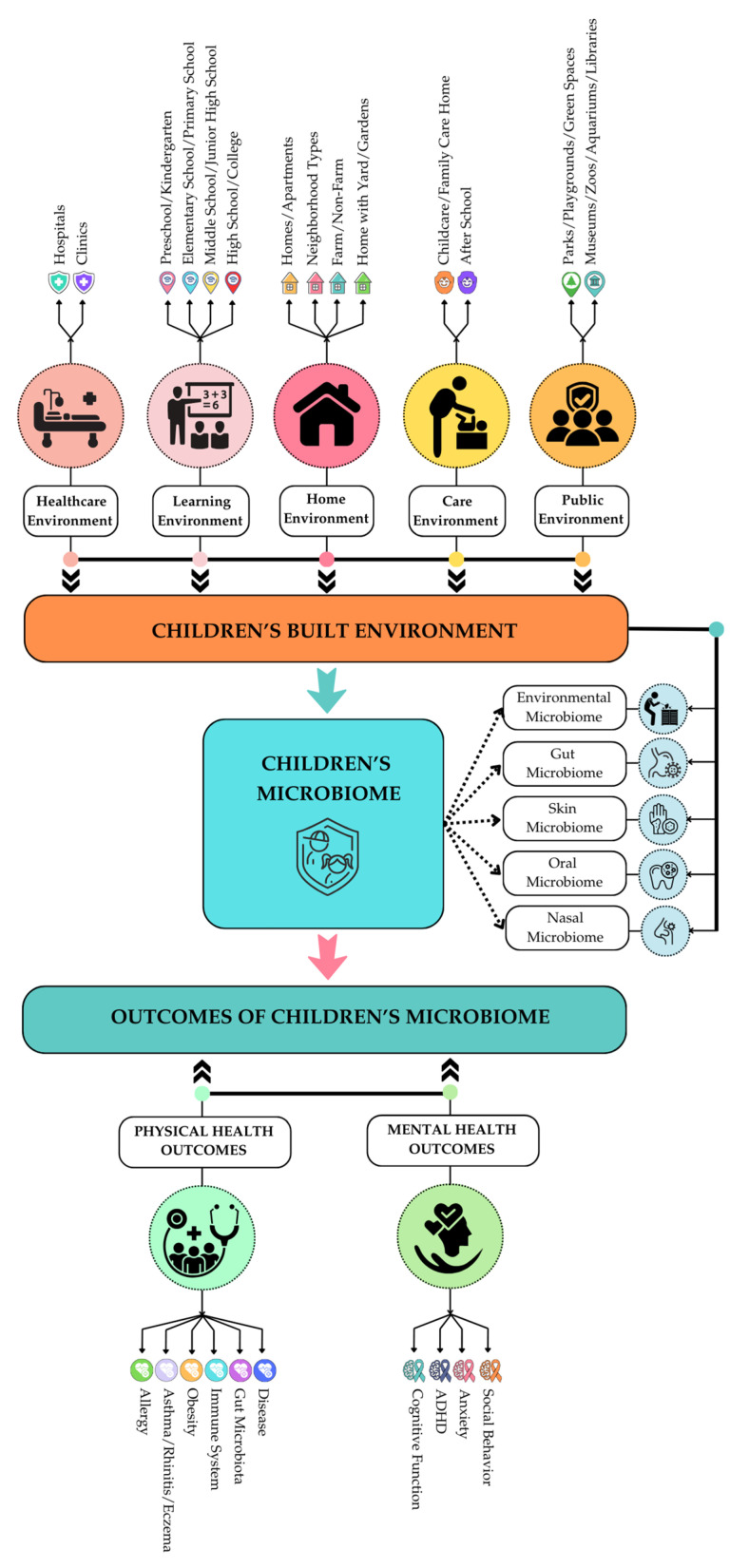
Comprehensive Diagram of Childhood Built Environment and Microbiome: The diagram shows how we classified major types of childhood BE, how the BE may influence microbiome, and how alteration in exposure to bacterial communities impacts physical and mental health outcomes.

Based on this complex spectrum of BE and its definitions, and potential impacts on children’s microbiomes, we developed our childhood BE context (Figure 1) to produce a holistic framework for the intended scoping review.

This diagram establishes our search (inclusionary and exclusionary criteria). With our intended goal to stay in the BE realm connected to BE design, we divided childhood BE into five types: healthcare environments, learning environments, home environments, care environments, and public environments. These BE categories (and subsequent sub-categories) capture all major places and spaces where children–BE interactions take place at different age points and influence children’s exposure to microbiota. We intentionally excluded temporal and behavioral BE aspects (e.g., consumption of antibiotics, breastfeeding habits, etc.) from the review. As mentioned previously, these temporal and behavioral BE aspects are influential in determining microbiome composition. However, they fall outside the scope of this review as they are not directly connected with or controlled by BE design characteristics or interventions. Some behavioral attributes, however, have causal connections with BE characteristics. For example, children’s behavior and activities associated with exposure to nature (dirt, soil, plants, and other organic materials, etc.) are afforded by the presence of natural elements in childhood BE, and, therefore, were not excluded from the review.

### 2.2. Inclusion and Exclusion Criteria

The microbiome of the built environment (BE) is affected by multiple variables. This review included factors such as natural and mechanical ventilation, environmental parameters (e.g., temperature, humidity, airflow, and air particles), building design, outdoor sources (e.g., soil, vegetation, water, land use, ecology, and urbanization levels), and indoor environments (air quality, indoor surfaces, indoor plants). We excluded most human factors (diet, medication, smoking habits, etc.), occupant-associated microbiomes, and population-based microbiome differences (e.g., human gut, oral cavity, and skin). As mentioned before (in Section 2.1), only certain child behaviors and activities associated with or influenced by BE were included.

The search targeted journal articles published between 2010 and 2024 (a 15-year period). The criteria for including studies are detailed in Table 1. A thorough review of 631 citation records (comprising articles from Databases 617 and Google Scholar 14) was conducted to screen for relevance based on predetermined inclusion and exclusion criteria. This process was crucial for identifying and selecting studies relevant to the research objectives.

### 2.3. Systematic Review Method

This systematic study was reported following the Preferred Reporting Items for Systematic Reviews and Meta-Analyses (PRISMA) principles, which were designed by a non-partisan scientific group and established guidelines for conducting and publishing systematic reviews and meta-analyses [29]. Ethical consent was not required because this was a secondary analysis of existing data.

Three sets of search terms were used across the following databases: PubMed, Scopus, Web of Science, and ProQuest. The title of this research was used to locate pertinent studies on Google Scholar. The search phrases were meticulously formulated by examining previously identified relevant articles’ titles, abstracts, and keywords. The Boolean operator “OR” was applied to differentiate the search words inside each set, while the operator “AND” combined several sets. The search terms are presented in Table 2 below.

### 2.4. Prisma Diagram

**Figure 2:**
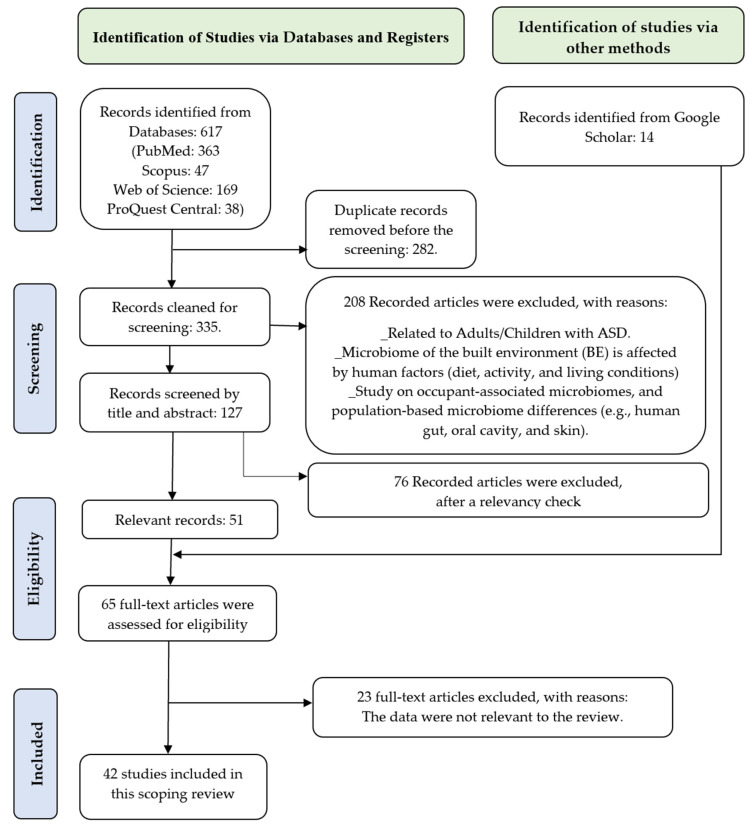
PRISMA 2020 flow diagram.

The literature search across four databases yielded 617 records (except Google Scholar), with the following unique citations from each source: 363 from PubMed, 47 from Scopus, 169 from Web of Science, and 38 from ProQuest Central. These records were imported into an EndNote library, where 282 duplicate entries were removed. Following the inclusion and exclusion criteria, an initial screening excluded 208. The remaining 127 full-text articles were assessed by reading the heading and abstract, and after a relevancy check, 51 journal articles were included. An additional 14 new studies were identified through a title search conducted on Google Scholar. Following a comprehensive full-text review of all 65 studies, 42 studies met the eligibility requirements and were included in the scoping review. This process adhered to the Preferred Reporting Items for Systematic Reviews and Meta-Analyses (PRISMA) guidelines, which were detailed in the PRISMA flow diagram (Figure 2).

## 3. Review Summary

### 3.1. Research Questions, Dependent, and Independent Variables

We first tried to understand from the reviewed studies (n = 42) what research questions guided them, what variables (dependent and independent) were measured, and whether it is possible to group the studies according to their core research questions and variables.

**Figure 3:**
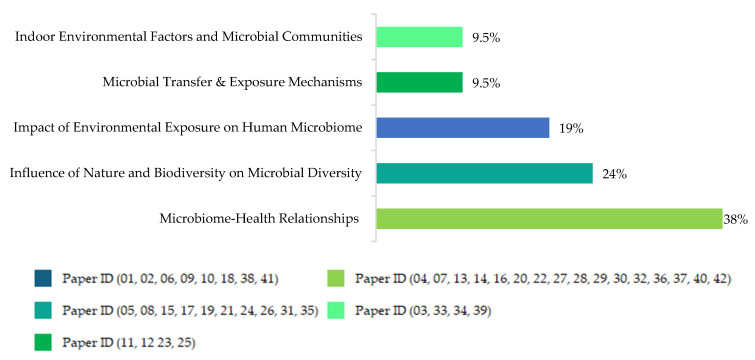
Thematic Streams of Research Questions.

The overarching question is exploring a three-way relationship between a) how BE characteristics can alter the BE microbiome, b) how the BE microbiome impacts children’s exposure to bacterial communities (skin, oral, fecal, or gut microbiota), and c) how exposure to and abundance (or lack) of certain bacterial communities are associated with child health and well-being outcomes (prevalence of certain diseases, immune system, behavioral outcomes, etc.). Some of the reviewed papers investigated all three segments of this complex relationship, but many investigated only one or two segments. We explored the research questions (and associated hypotheses) in these papers for our initial understanding of how they approached investigating the relationship(s). Journal articles usually do not explicitly state their research questions, so we framed them in our language based on their investigations. This review paper identified five thematic streams (Figure 3) that correspond to the research questions in the 42 reviewed studies (Appendix A).

Figure 3 shows a concise thematic breakdown of the research questions addressed across the reviewed literature. The dominant focus lies in microbiome–health relationships (38%), reflecting a strong research orientation toward understanding the role of microbial communities in human health and disease. This is followed by investigations into the influence of nature and biodiversity on microbial diversity (24%) and the impact of environmental exposure on the human microbiome (19%). In contrast, there is a lack of studies to identify how contact with built environments affects the transfer and diversity of microbial communities.

Table 3 below categorizes the reviewed studies and identifies the primary research questions. Each cell represents a study (identified by its Paper ID) that addresses a particular research question. This visual matrix reveals the recurring foci such as the effects of environmental exposure on the human microbiome (e.g., Paper IDs 1, 2, 6, 9, 10, 18, 38, 41) and the influence of nature and biodiversity on microbial diversity (e.g., Paper IDs 5, 8, 15, 17, 19, 21, 24, 26, 31, 35). Several studies share common research questions, such as how exposure to biodiversity-enriched/natural environments affects children’s microbiota. Although some studies share multiple themes, we selected each paper for its category based on the area it emphasizes most.

Simultaneously, Table 4 complements Table 3 by providing a variable-level mapping of the reviewed studies. It distinguishes between independent variables (e.g., environmental context, microbial diversity, human behaviors) and dependent variables (e.g., health outcomes, microbial composition, cognitive effects). The matrix illustrates how certain variables occur across thematic streams. For instance, “environmental exposures” (e.g., soil, urban green spaces, pollutants) and “microbial diversity and composition” are frequently measured in studies under the environmental exposure and biodiversity influence streams. Similarly, health-related outcomes, such as allergies, respiratory infections, and immune responses, are central to the microbiome–health relationships stream.

### 3.2. Study Characteristics of the Reviewed Studies

**Figure 4:**
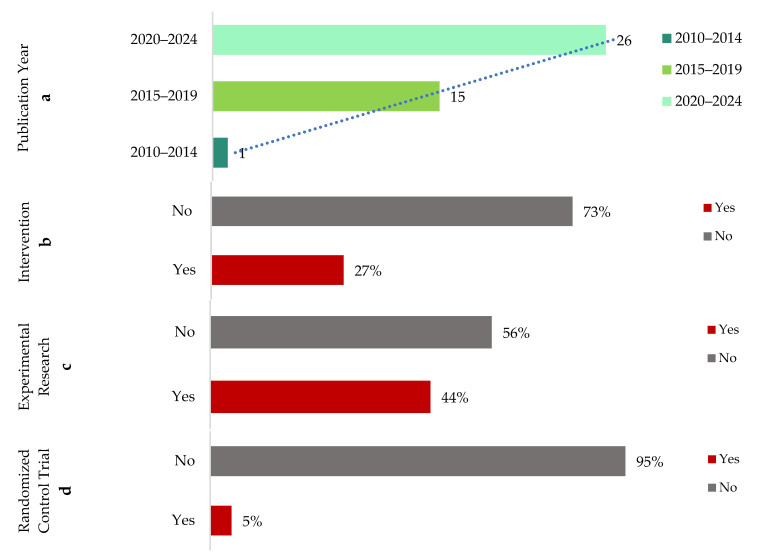
Study Characteristics of Reviewed Studies ((**a**) publication year, (**b**) intervention, (**c**) experimental research, and (**d**) randomized control trial).

The literature search targeted journal articles published over a 15-year period from 2010 to 2024. The analysis (Figure 4a) revealed an exponential growth in publications on the reviewed topic during this timeframe. Specifically, only one article (2.4%) was published between 2010 and 2014, while the publication rate increased to 15 articles (35.7%) between 2015 and 2019. The most significant growth occurred recently, with 26 articles (61.9%) published from 2020 to 2024. This temporal trend underscores that research examining the impact of built environment elements on children’s microbiomes has undergone rapid expansion, reflecting a growing scholarly interest in recent years. Notably, the onset of the COVID-19 pandemic in early 2020 served as a significant catalyst for increasing scholarly focus in this field. The heightened awareness of health, hygiene, and environmental influences resulting from the pandemic likely contributed to the accelerated growth of research exploring how built environmental factors influence children’s microbiomes.

Most reviewed studies (73%) did not include interventions (Figure 4b), indicating a predominantly correlational research approach. Experimental research methods were used in nearly half (44%) of the studies (Figure 4c), while slightly more than half (56%) adopted non-experimental designs. Furthermore, randomized control trials (RCTs), a rigorous experimental approach, were infrequently utilized, with only 5% of studies employing this method (Figure 4d). These findings collectively demonstrate that current research predominantly employs observational and correlational methodologies, pointing to a limited but emerging use of experimental and intervention-based designs.

### 3.3. Contextual Characteristics of the Reviewed Studies

**Figure 5:**
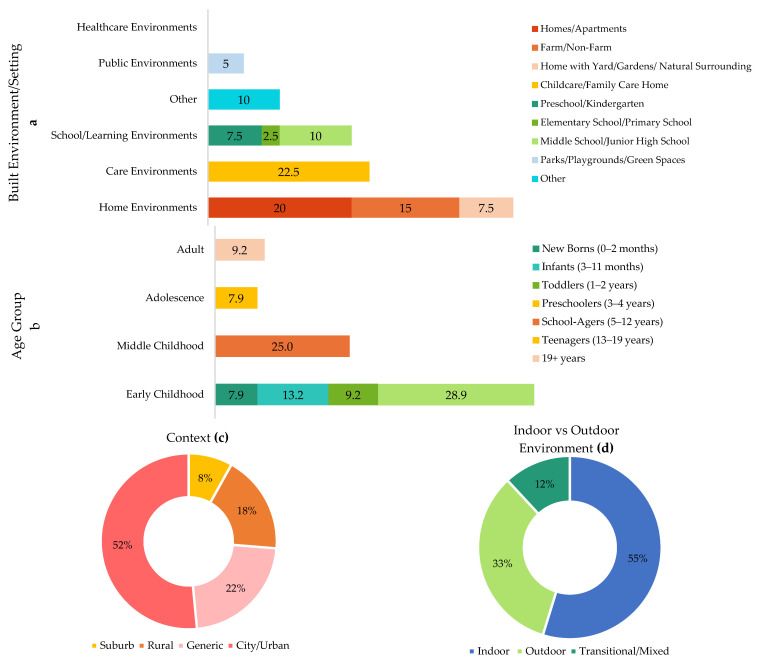
Contextual Characteristics of the Reviewed Studies ((**a**) built environment/setting, (**b**) age group, (**c**) context, and (**d**) indoor vs. outdoor environment).

The reviewed studies predominantly focused on home environments (42.5%), with a particular emphasis on residential settings, including homes, apartments, farms, and homes with yards or natural surroundings, highlighting the importance of proximity to nature in influencing children’s microbiome diversity (Figure 5a). Care environments, specifically childcare and family care homes, also constituted a significant research focus (22.5%), underlining their critical role in shaping microbial exposure during early childhood. School and learning environments comprised 20% of the studies, with preschool and middle school settings being most frequently investigated. Conversely, public environments, including parks, playgrounds, and green spaces, accounted for only a small proportion (5%) of investigations, despite their recognized potential benefits for microbial diversity. Notably, healthcare environments were entirely absent from the reviewed studies, indicating a gap that warrants further investigation.

Although this review primarily focuses on childhood BE, some studies included broader age ranges, encompassing teenagers and adults alongside younger children; thus, those older age groups (Figure 5b) were also considered in the analysis. Early childhood was the most extensively researched category, collectively accounting for 59.2%. Middle childhood (school-age children, 5–12 years) was also substantially studied, accounting for 25% of the research, whereas adolescents (teenagers, 13–19 years) accounted for 7.9%. Adults (19+ years) appeared in 9.2% of the studies, reflecting cases where studies involved both children and adults. This age-group distribution highlights a research emphasis on early and middle childhood as critical developmental periods for microbiome diversity.

However, the reviewed studies predominantly focused on urban or city contexts (52%), with suburban areas receiving the least attention (8%) (Figure 5c). Regarding environmental typology (Figure 5d), indoor settings were most frequently examined (55%), highlighting a significant research interest in indoor built environments as critical sites for microbial exposure.

### 3.4. Understanding Children’s Well-Being: The Role of the Microbiome

When we refer to the well-being of children, we are considering their overall health, which includes both physical and mental aspects, and the environmental factors that contribute to this state of health. In contemporary research, well-being is often tied to a child’s immune system function and ability to fight off diseases, whether environmental allergies, asthma, or even more serious conditions like childhood leukemia and cancers. Studies on microbiomes and the microbial community living in and on the human body suggest that exposure to a diverse range of microbes, particularly in early life, plays a crucial role in shaping immune responses and health outcomes. The understanding of children’s well-being in these studies hinges on the premise that microbiomes can protect against environmental stimulations by training the immune system to respond appropriately to allergens and pathogens.

The concept of immune tolerance is central in these studies. Specifically, researchers frequently investigated how exposure to microbes in early childhood can help the immune system learn to distinguish between harmful invaders (like pathogenic viruses and bacteria) and harmless environmental components (like pollen or food proteins, or non-pathogenic microorganisms). This concept is important concerning the rising prevalence of allergies, autoimmune diseases, and other immune-related conditions, which are increasingly common in children exposed to urbanized, hygienic environments [30]. In many of these studies, researchers have explored how microbiomes may influence the development of conditions such as asthma, allergic rhinitis, childhood cancers, and autoimmune diseases by modulating immune responses and microbial diversity.

**Figure 6:**
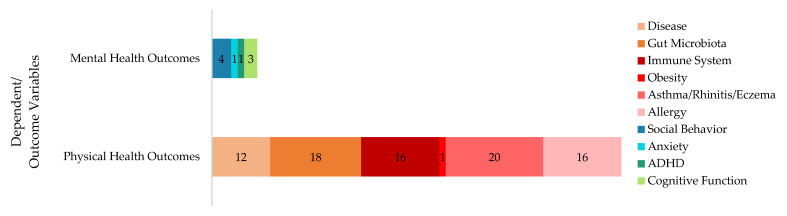
Dependent/outcome variables of the reviewed studies.

Figure 6 visually represents the focus of the reviewed studies on various health outcomes to understand the role of microbiomes in children’s well-being. Specifically, it reveals that a substantial number of studies, at 18% and 20%, investigated gut microbiota concerning asthma, rhinitis, and eczema, respectively, underscoring significant research interest in these areas. The immune system also garnered considerable attention, with 16% of studies, similar to those on allergies. Notably, mental health aspects such as anxiety (11%) and social behavior (4%) indicate a growing interest in the psychological impacts associated with microbiome studies. This distribution underscores a stronger emphasis on physical health in the current research landscape, with mental health aspects being less explored.

### 3.5. Microbiome Samples and Sampling Methods

Most studies reviewed here investigated the microbiomes present in children’s environments, primarily focusing on dust, fecal samples, skin, saliva, and air samples. Each of these samples offers unique insights into different microbial communities and their potential impact on health. Though some researchers investigated a single type of sample (dust or feces), many of the studies applied different combinations of samples based on the objective of their research. We have found ten different combinations of sampling in these studies, as shown in Table 5.

#### 3.5.1. Dust Samples

Dust was one of the most frequently analyzed samples in microbiome studies, especially concerning environmental exposures. Dust can contain a rich diversity of environmental microbes that children are exposed to in their homes, daycare centers, and schools. In total, 51.3% of the research included dust along with other samples as the primary source of microbial DNA, whereas 43.2% of them were exclusively dust. Dust from indoor environments was used to explore microbial exposure and its effects on children’s immune health, particularly concerning asthma and other respiratory conditions [31,32,33]. Dust is a valuable sample because it provides insight into ambient microbial communities, including bacteria, fungi, and other microbial entities, that interact with children’s immune systems. The advantage of using dust is that it provides a broad picture of the microbial environment a child is exposed to over time, is non-invasive, and is easy to collect.

#### 3.5.2. Fecal Samples

Fecal samples were used extensively in studies that focused on the gut microbiome. The gut is home to trillions of microorganisms, and its diversity is a critical factor in immune development. Fecal samples were collected to study the gut microbiome in children living in different environments (e.g., rural vs. urban) and to understand the link between gut microbes and disease prevention, particularly concerning autoimmune diseases like asthma and allergies [34,35,36]. The fecal sample was also used to study how various personal, environmental, and dietary factors correlate with the diversity and composition of children’s gut microbiota [37]. Fecal samples are key because they reflect the microbial community that directly influences the immune system, but they are invasive, which can be a limitation when attempting to sample large populations.

#### 3.5.3. Skin and Saliva Samples

Oral sample microbiome showed significant changes in children whose parents had detectable levels of azinophos-methyl (AZM), a type of pesticide used in farms, in their blood [38]. To study whether the direct contact with soil and plant material changed the skin microbiota, skin swabs were used as a source of microbial DNA, and it was found that even short-term direct interaction with soil can alter different phyla of bacteria such as acidobacteria, actinobacteria, proteobacteria, and so on [39]. The skin and saliva microbiomes can be sampled less invasively than fecal samples, making them appealing for large-scale studies.

#### 3.5.4. Air Samples

In addition to microbial samples from bodily surfaces, air samples were used in several studies to assess the impact of air pollutants on children’s microbiomes. To **analyze** airborne microbiomes and study how environmental air pollution affects microbial composition and children’s respiratory health, airborne microbiomes were used as microbial DNA sources [40]. Air samples provide insight into the external microbial exposure children experience and how pollutants might disrupt the microbiome and lead to diseases like asthma.

### 3.6. Biological Experiments

#### 3.6.1. Molecular Analysis

For sequencing microbial DNA, Illumina MiSeq was the most common method used by the researchers, along with PacBio and Ion Torrent. Most researchers used 16S rRNA sequencing, which sequences the diverse bacterial community present in the biological samples. Though very few studies checked only the fungal community with the ITS sequencing method [41], most conclusive studies incorporated both 16S rRNA and ITS sequencing to understand the diversity and richness of both bacterial and fungal communities [31,32,42,43,44]. Some studies also incorporated a qPCR (Quantitative Polymerase Chain Reaction) to measure the microbial load in various samples [32,39,40,42,44,45]. The qPCR is a highly sensitive and specific method for measuring microbial concentrations, and it is particularly useful when examining the relationship between microbial load and health outcomes like asthma or allergies.

#### 3.6.2. Metabolomic Analysis

The inclusion of metabolomics adds a significant layer of understanding because it not only looks at microbial diversity but also at the metabolites produced by microbes, which can have direct biological effects on the immune system. Metabolomics helps connect microbial community changes to health outcomes by identifying bioactive compounds that might influence immune function or contribute to diseases like asthma. The value of metabolomic studies lies in their ability to reveal functional aspects of microbial communities: how microbes may affect health beyond just their presence or absence. These studies provide a deeper understanding of the microbial–immune interactions that influence disease outcomes. Though most studies used LC-MS (Liquid Chromatography-Mass Spectrometry) to study the metabolites present in the biological sample [32,37,46,47], some studies used GC-MS (Gas Chromatography) to study the volatile metabolites [38,48]. Researchers who studied the association of environmental factors influencing gut microbiome and allergy response used a biochemical method, Phadiatop (ThermoFisher Scientific, Waltham, MA, USA), to check the Immunoglobulin E (IgE) concentration in the blood serum, which is an indicator for allergic response, in addition to microbial diversity [49,50]. Another dimension of this field was added when scientists used a Semi-Volatile Organic Compound (SVOC) biomarker and serum IgE level to study how SVOC exposure from the indoor environment in everyday life alters children’s microbiota as well as their atopic disease response using urine, blood, and fecal samples [51]. While these studies offer valuable insights, they are also more complex and require more advanced analytical tools, software, and expertise, making them more resource-intensive than basic sequencing methods.

### 3.7. Bioinformatic and Metabolomic Data Analysis: Tools and Software

The tools used for analyzing sequencing data and metabolomics are diverse, with each study choosing methods that best align with its research goals. Common tools for sequence cleanup include Trimmomatic and Cutadapt (Germany), which are used to remove low-quality sequences and adapters from raw data before analysis [33,46,50]. For alignment and taxonomic annotation, tools like QIIME2 [35,37,40,47,52], Mohur [36,39,53], and UPARSE are widely used, with the QIIME2 pipeline (USA) being the most advanced and versatile for microbiome analysis. These platforms enable the processing of 16S rRNA and ITS sequencing data, providing insights into microbial diversity and community composition. QIIME2 (Quantitative Insights into Microbial Ecology2) is a bioinformatic platform which allows the filtering and cleaning up of the sequence reads, clustering sequences into ASVs (Amplicon Sequence Variants) or OTUs (Operational Taxonomic Unites), classifying sequences into taxonomic groups, analyzing within and between sample diversity metrics (alpha and beta diversity), and identifying differences in microbial communities across samples. This is a comprehensive metagenomic data analysis pipeline and an open-access resource, making it the most popular among researchers all around the world.

For metabolomics data, tools like XCMS (Scripps Research, San Diego, CA, USA) and MZmine (OIST, Onna, Japan) are commonly used for data processing, with LC-MS data being analyzed through specialized software for peak detection, alignment, and compound identification. METLIN (Scripps Research, USA), mzCloud (HighChem Ltd., Bratislava, Slovakia), and the Human Metabolome Database (University of Alberta, Edmonton, AB, Canada) are used [47,48]. These tools are essential for linking specific metabolites to health outcomes and understanding the biochemical pathways involved in microbial–host interactions.

## 4. Discussion

### 4.1. An Emphasis on Children’s Exposure to Nature

**Figure 7:**
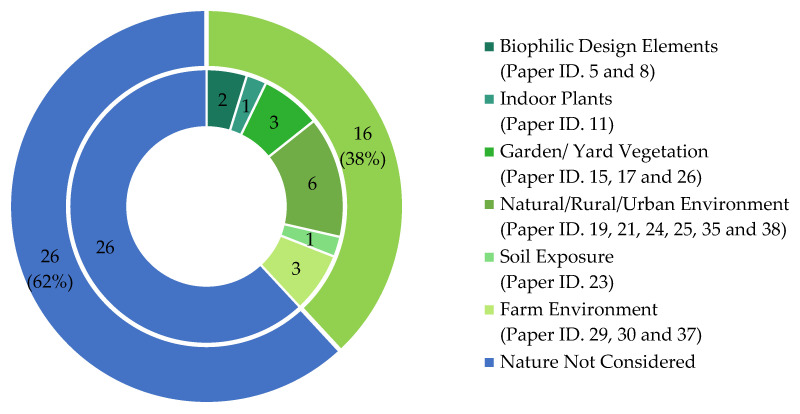
The Ratio of Reviewed Studies Considering Nature as an Indicator.

One of the most notable discussion points in this review is an emphasis on children’s exposure to nature and its potential microbial benefits to BE. In total, 38% of studies (16 out of 42) directly or indirectly investigated nature’s role in altering childhood microbiomes (Figure 7).

The reviewed papers covered a wide range of nature interventions and their subsequent effects on childhood microbiomes, including biophilic design elements [23,40], presence and amount of indoor plants [54], garden or yard vegetation [36,53,55], farm (vs. non-farm) living environments [56,57], etc. Children’s exposure to nature has gained significant attention over the last decade, especially in the face of a changing childhood landscape with growing screen time, lack of physical activity, and rapidly diminishing time spent outdoors. The benefits of children’s exposure to nature are well-documented by research, including reduced risks of myopia, reduced stress, increased physical activity, and attention restoration. The pieces of evidence presented in this review that nature intervention can also alter microbiomes for positive health advancement are a bold statement supporting the children and nature movement. However, investigating nature’s role in altering childhood microbiomes is more complex. First, there are too many variables and complex mechanisms that contribute to childhood microbiomes. Controlled experiments, therefore, are extremely challenging. Only 5% of all studies (n = 42) reviewed in this paper claimed to have conducted randomized controlled trials (RCTs)—the gold standard of experimental research (Figure 4d). Only one study by Sobko and colleagues [35] claimed to conduct an RCT to determine how nature exposure impacts children’s gut health and psychological well-being. The study claimed to be the first one of its kind to demonstrate the impact of nature-related activities on the gut microbiota, fecal serotonin, and psychosocial behavior of preschool children. A closer look reveals that the study included 27 children in the intervention group (‘Play&Grow’ intervention) and 18 children in the control group. Little information was provided about ‘Play&Grow’, which was described as a “a program to reconnect preschoolers to nature and induce changes in health behaviors and outcomes by having outdoor activities that promote exposure to nature”. Yet, how potential confounding factors (home diet, home nature exposure, intakes of antibiotics during study period, etc.) were eliminated is vaguely described as a ”reminder to the participants to certain resources”. It is obvious that the level of control in this study, which relied on participants’ ability to alter/track habits based on some resources, was weak. Properly conducted RCTs are needed in this investigation, but future research initiatives should acknowledge the challenges of conducting RCTs to measure nature’s impact in altering childhood microbiomes.

### 4.2. Behavioral Outcomes of BE Microbiome

Nine papers (Figure 6) were identified that highlighted the impact of BE in altering microbiomes that are influential for mental health and behavioral outcomes. The BE-influenced microbiome and its effect on children’s behavioral outcomes and mental well-being have garnered attention. In the Sobko et al. study (2020) [35], a reduction in perceived stress, particularly anger, was observed in children participating in the intervention group (IG) compared to the control group (CG). Dockx et al. (2023) [31] found that a higher fungal diversity in indoor dust was associated with better behavioral and cognitive outcomes, including lower hyperactivity and improved attention and psychomotor speed in children. One study [58] investigated the association between indoor microbial diversity in the home environment early in life and hyperactivity/inattention symptoms in children at ages 10 and 15. They found that a higher bacterial diversity early in life was inversely associated with hyperactivity/inattention at age 10, while fungal diversity showed a direct association. The above studies are connected with a growing body of evidence and understanding that the gut microbiome is not only an indicator but also a bi-directional influencing factor on behavioral outcomes.

### 4.3. Rare Connection to BE Design

Since most of the reviewed papers come from the fields of medical or biological sciences, very few papers discussed the ‘design’ aspects of the BE that may affect children’s microbiomes. One paper [23] extensively discussed detailed BE design considerations and focused on using the Microbiome-Inspired Green Infrastructure (MIGI) principles to design outdoor environments that promoted microbiome diversity, which is critical for childhood development, immune health, and overall well-being. Two papers mentioned ‘biophilic design’ [40,59]: a concept used within the building industry to increase occupant connectivity to the natural environment using direct nature, indirect nature, and space and place conditions. Future research should investigate biophilic design interventions and alterations in childhood microbiomes.

### 4.4. Was Multiple Sampling Necessary? Is Metabolomics Worth It?

The studies reviewed explored a variety of sampling strategies, including skin, saliva, nose swab, fecal, blood, urine, potting soil, and air samples. The question remains: did multiple sampling methods provide a more comprehensive understanding of the microbiome’s role in children’s health, or is a single sample sufficient to answer broader questions?

Multiple sampling methods (feces, saliva, skin, and air) do provide a holistic view of microbial exposure and its potential impact on health. However, it is not always necessary to use multiple sampling strategies to answer specific research questions. For example, a fecal sample alone can provide comprehensive information about gut microbiomes, which play a significant role in immune development. However, for studies focused on respiratory health, combining skin and air samples may be crucial for understanding the external microbial exposure children face.

**Figure 8:**
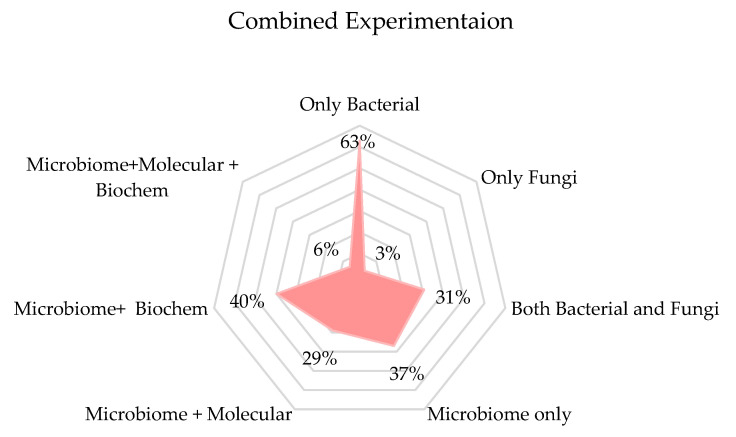
Combination of Biological Experimentations (Microbial, Biochemical, and Molecular) Utilized to Understand the Impact of Microbiome on Children’s Health. The radar chart demonstrates a pronounced emphasis on “Only Bacterial” experiments, which constitute 63% of the reviewed studies, indicating a substantial focus on bacterial experimentation exclusively. Conversely, categories such as “Only Fungi” and “Microbiome + Molecular + Biochem” are markedly less emphasized, capturing merely 3% and 6% of the studies, respectively. This distribution suggests these areas are less prioritized within the research framework.

As for the metabolomic analysis, while it offers deep insights into microbial functions and immune modulation, its high cost and complexity make it unnecessary for all studies. However, when investigating the functional impact of microbes on immune health, especially regarding diseases like asthma, metabolomics can provide invaluable data that sequencing alone cannot. Figure 8 shows that 63% of the research we discussed studied only bacterial community, whereas only 6% of these studies had combinations of microbial, molecular, and biochemical experimentations to understand the contribution of the microbiome in children’s health. To have a comprehensive understanding of the impact of microbial diversity in children’s microbiomes, we believe that a comprehensive study of both bacterial and fungal communities along with metabolic profiling is important.

### 4.5. Future Research Directions for Children’s Built Environment and Microbiome

To advance this emerging field, future research should focus more on how specific architectural and/or landscape features (e.g., natural ventilation, courtyards, green roofs, and biophilic elements) affect children’s microbial exposures and associated health outcomes. Such studies could guide evidence-based design strategies that promote beneficial microbiome development. One surprising finding of this review is the notable lack of research focused on healthcare environments (Figure 5a). This gap may stem from the common perception of these settings as inherently ‘sterile’, where microbial diversity is assumed to be minimal or undesirable. The role of healthcare environments, including hospitals and clinics, deserves attention, especially during critical developmental windows when microbiomes are most impactful and malleable. Understanding how these high-contact settings shape microbial diversity could inform infection control and health promotion practices tailored for pediatric populations. Furthermore, there is a need to explore how behavioral factors, like outdoor play, nature-connectedness, and dietary habits, interact with spatial and temporal dimensions of the built environment to influence children’s microbiomes. Longitudinal, interdisciplinary studies integrating environmental design, behavioral science, and microbiology will be key to developing holistic frameworks for designing health- and microbiome-supportive environments for children.

## 5. Conclusions

This scoping review highlights the growing recognition of the built environment as a crucial determinant of children’s microbiome composition, exposure to diverse microbiota, and subsequent health and well-being outcomes. Despite increasing interest in this intersection, significant research gaps remain. Future studies must adopt interdisciplinary approaches that integrate microbiology, environmental science, landscape architecture, and public health to comprehensively assess how built environments shape microbial exposures in early childhood.

Beyond the fields associated with the built environment, the findings of this review have important implications across multiple health-related fields, including pediatrics, epidemiology, environmental health, and preventive medicine. This review offers valuable insights for guiding early-life interventions aimed at strengthening immune development and mental well-being and informs preventive strategies aimed at reducing the risk of allergies, asthma, and behavioral disorders. It also has the potential to shape public health policies, support pediatric healthcare planning, and promote nature-integrated design practices that prioritize microbial diversity as a pillar of healthy childhood development.

## Figures and Tables

**Table 1:** Inclusion and Exclusion Criteria.

Inclusion Criteria	Exclusion Criteria
1. Articles published from 2010 to 2024	1. Unavailable full text
2. English language	2. Focus on children with Autism Spectrum Disorder (ASD)
3. Focus on the built environment	3. Not related to the human factor and/or occupant factor
4. Focus on children	4. Study with adults

**Table 2:** Search Keywords and Search Terms.

Children	Child * OR Early child * OR Preschool * OR Kid OR Kindergarten OR Young child * OR School-aged child * OR Youth
Microbiome	Microbial OR Microbiology OR Microorganism * OR Microbe *
Built Environment	Outdoor OR Nature * OR Veg * OR Indoor OR Surface OR Childcare OR Home OR Land * OR Physical * OR Environment * OR Soil OR Air OR Water OR Bio * OR Rural OR Urban OR Build * OR Expos * OR Vent *

Note. The asterisk “*” is a truncation symbol that directs the search engine to find all forms of a given word.

**Table 3:** Mapping of Research Questions Across Studies.

STUDY TOPIC & PAPER ID	1	2	3	4	5	6	7	8	9	10	11	12	13	14	15	16	17	18	19	20	21	22	23	24	25	26	27	28	29	30	31	32	33	34	35	36	37	38	39	40	41	42
**1. Impact of Environmental Exposure on Human Microbiome**																																										
What is the variation between microbial diversity of taxa between home and daycare environments?																																										
How does pesticide exposure influence the oral microbiome composition of the children of farmworkers?																																										
How do indoor microbiome and metabolites influence the diversity of the gut microbiota in children??																																										
How do environmental exposures, both indoor and outdoor, influence the microbiome composition in children?																																										
How does the living environment (rural vs. urban) influence the composition and diversity of skin microbiota?																																										
**2. Influence of Nature and Biodiversity on Microbial Diversity**																																										
How does the composition of the airborne microbiome in indoor environments influenced by biophilic design?																																										
How do nature-based solutions influence microbiome diversity in early childhood educational settings?																																										
How does exposure to biodiversity-enriched/ natural environments affect children’s microbiota?																																										
What are the impacts of outdoor nature-related activities on gut microbiota and psychological health of children?																																										
How does direct contact with soil/plant materials altering skin microbiota?																																										
**3. Microbial Transfer & Exposure Mechanisms**																																										
Can indoor plant leaves and soil bacteria temporarily establish themselves on human skin after physical contact?																																										
How does the microbial composition vary between different surfaces and locations within childcare centers?																																										
How do children’s behaviors and soil pollution contribute to their exposure to soil contaminants?																																										
How does exposure to urban green spaces affect the transfer and diversity of environmental microbes to human skin?																																										
**4. Microbiome-Health Relationships (Respiratory, Immune, Allergies, Asthma)**																																										
How are microbiome variations associated with allergic and non-allergic rhinitis symptoms among students?																																										
How do the profiles of indoor metabolites and chemicals differ between homes with/without asthma or allergic rhinitis?																																										
How does exposure to thirdhand smoke (THS) alter the microbiomes of children and their home environments?																																										
How do indoor microbiome composition and environmental factors influence the respiratory microbiota/asthma/allergy?																																										
How does SVOC exposure affect gut microbiome and dysbiosis?																																										
How does the Indoor microbiome diversity influence asthma/ hyperactivity/ inattention symptoms?																																										
How does exposure to PAHs affect the commensal microbiota of children and disrupt endocrine signaling pathways?																																										
How does exposure to farm environments influencing microbiome and asthma risks?																																										
**5. Indoor Environmental Factors and Microbial Communities**																																										
How does the diversity of fungal microbiota in indoor dust relate to cognitive and behavioral outcomes in children?																																										
How do indoor emissions and outdoor ventilation contribute to the microbial communities?																																										
How does the microbial community composition in the dust of a newly opened kindergarten change over time?																																										
How do human occupancy and seasonal changes influence airborne microbiota in daycare centers?																																										

In this table, the study topics of each paper are color-coded as follows: 1. Impact of Environmental Exposure on Human Microbiome = 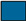, 2. Influence of Nature and Biodiversity on Microbial Diversity = 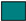, 3. Microbial Transfer & Exposure Mechanisms = 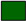, 4. Microbiome-Health Relationships (Respiratory, Immune, Allergies, Asthma) = 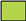, 5. Indoor Environmental Factors and Microbial Communities = 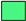.

**Table 4:** Mapping of Independent and Dependent Variables Across Thematic Streams of Research Questions.

THEMATIC STREAMS OF RESEARCH QUESTIONS AND THEIR ASSOCIATED PAPER IDs	**INDEPENDENT VARIABLES**																													**DEPENDENT VARIABLES**														
	Environmental contexts:								Microbial diversity and composition:			Environmental exposure:								Human Activities and Behaviors:					Temporal/spatial dynamics:					Health and disease outcomes:					Microbial diversity and composition:						Cognitive and behavioral outcomes:			
	Home vs. daycare	Farm vs. non-farm	Nature-oriented vs. conventional daycare	Urban vs. rural environment	Indoor vs. outdoor environments	Nature-based solutions/ Biodiversity intervention	Indoor plants (leaves and soil)	Gardening families vs non-gardening families	Bacterial/fungal/ microbial communities	Dust microbiota	Surface-specific microbiota	Semivolatile organic compounds (SVOCs)	Pesticides (e.g., azinphos-methyl)	Polycyclic aromatic hydrocarbons (PAHs)	Outdoor/indoor air pollutants (e.g., NO_2_)	Indoor relative humidity	Urban green	THS exposure	Soil pollution	Dietary habits	Occupational Status	Pet exposure	Personal hygiene practices	Children’s behaviors	Seasonal changes	Spatiotemporal variation	Occupant density/ Room usage	Ventilation rates	Time and duration of exposure	Allergic and non-allergic rhinitis (AR/NAR)	Asthma, Eczema, Respiratory infections	Immune health	Psychological well-being	Antibiotic resistance genes (ARGs)	Gut, Oral, Skin, Nasal, airway microbiota	Indoor and outdoor microbial community	Dysbiosis (unhealthy microbial balance)	Non-pathogenic environmental mycobacteria	Human-associated microbial taxa	Fungal/ Bacterial community composition	Cognitive function	Hyperactivity/inattention	Behavioral outcomes in children	Psychobehavioral development
Impact of Environmental Exposure on Human Microbiome (01, 02, 06, 09, 10, 18, 38, 41)																																												
Influence of Nature and Biodiversity on Microbial Diversity (05, 08, 15, 17, 19, 21, 24, 26, 31, 35)																																												
Microbial Transfer & Exposure Mechanisms (11, 12 23, 25)																																												
Microbiome-Health Relationships (Respiratory, Immune, Allergies, Asthma) (04, 07, 13, 14, 16, 20, 22, 27, 28, 29, 30, 32, 36, 37, 40, 42)																																												
Indoor Environmental Factors and Microbial Communities (03, 33, 34, 39)																																												

In this table, the thematic streams of research questions are color-coded as follows: 1. Impact of Environmental Exposure on Human Microbiome = 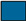, 2. Influence of Nature and Biodiversity on Microbial Diversity = 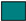, 3. Microbial Transfer & Exposure Mechanisms = 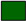, 4. Microbiome-Health Relationships (Respiratory, Immune, Allergies, Asthma) = 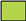, 5. Indoor Environmental Factors and Microbial Communities = 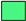.

**Table 5:** Ten Different Combinations of Multiple Sampling Strategies Researchers Adopted to Collect Microbial DNA (Each unique color refers to a unique type of sample combination).

Combined Sample	Sample Types											
	Dust	Feces	Soil	Nose	Skin	Mouth	Ear	Urine	Blood	Air	Leaf	Potting soil
Type 01												
Type 02												
Type 03												
Type 04												
Type 05												
Type 06												
Type 07												
Type 08												
Type 09												
Type 10												

## Data Availability

Data sharing is not applicable (Review Paper).

## Appendix A

**ID**	**Paper Title**	**Citation**
1	Environmental microbiome in the home and daycare settings during the COVID-19 pandemic, and potential risk of non-communicable disease in children	[33]
2	Alteration of oral microbiome composition in children living with pesticide-exposed farm workers	[38]
3	Association of indoor dust microbiota with cognitive function and behavior in preschool-aged children	[31]
4	Associations between environmental characteristics, high-resolution indoor microbiome, metabolome and allergic and non-allergic rhinitis symptoms for junior high school students	[32]
5	Diversity and compositional differences of the airborne microbiome in a biophilic indoor environment	[40]
6	Impact of environmental characteristics on children’s gut microbiota—A pilot study in assessing the role of indoor microbiome and metabolites	[37]
7	Indoor metabolites and chemicals outperform microbiome in classifying childhood asthma and allergic rhinitis	[47]
8	Optimising Early Childhood Educational Settings for Health Using Nature-Based Solutions: The Microbiome Aspect	[23]
9	Impact of the environment on the microbiome	[1]
10	Indoor microbiome, air pollutants and asthma, rhinitis and eczema in preschool children—A repeated cross-sectional study	[52]
11	Temporary establishment of bacteria from indoor plant leaves and soil on human skin	[54]
12	The bacterial community of childcare centers: potential implications for microbial dispersal and child exposure	[60]
13	Altered microbiomes in thirdhand smoke-exposed children and their home environments	[46]
14	Associations between the indoor microbiome, environmental characteristics and respiratory infections in junior high school students of Johor Bahru, Malaysia	[42]
15	Environmental Influences on the Human Gut Microbiota: A Longitudinal Pilot Study	[55]
16	Exposures to Semivolatile Organic Compounds in Indoor Environments and Associations with the Gut Microbiomes of Children	[51]
17	Long-term biodiversity intervention shapes health-associated commensal microbiota among urban day-care children	[53]
18	Spatiotemporal variation of the indoor mycobiome in daycare centers	[41]
19	Biodiversity intervention enhances immune regulation and health-associated commensal microbiota among daycare children	[59]
20	Environmental shaping of the bacterial and fungal community in infant bed dust and correlations with the airway microbiota	[61]
21	Impact of outdoor nature-related activities on gut microbiota, fecal serotonin, and perceived stress in preschool children: the Play&Grow randomized controlled trial	[35]
22	Indoor microbiome, environmental characteristics and asthma among junior high school students in Johor Bahru, Malaysia	[45]
23	Mechanisms of children’s soil exposure	[21]
24	Natural environments in the urban context and gut microbiota in infants	[62]
25	Transfer of environmental microbes to the skin and respiratory tract of humans after urban green space exposure	[63]
26	Yard vegetation is associated with gut microbiota composition	[36]
27	Early life home microbiome and hyperactivity/inattention in school-age children	[58]
28	Endocrine disruption and commensal bacteria alteration associated with gaseous and soil PAH contamination among daycare children	[48]
29	Evaluating the Effects of Farm Exposure on Infant Gut Microbiome	[34]
30	Farm-like indoor microbiota in non-farm homes protects children from asthma development	[56]
31	Short-term direct contact with soil and plant materials leads to an immediate increase in diversity of skin microbiota	[39]
32	Thinking bigger: How early-life environmental exposures shape the gut microbiome and influence the development of asthma and allergic disease	[24]
33	From Homes to Schools—The Impact of Ventilation and Cleaning on the Bacterial and Fungal Ecology of the Built Environment	[43]
34	Longitudinal development of the dust microbiome in a newly opened Norwegian kindergarten	[44]
35	Nature-oriented daycare diversifies skin microbiota in children—No robust association with allergies	[49]
36	The classroom microbiome and asthma morbidity in children attending 3 inner-city schools	[64]
37	Environmental and mucosal microbiota and their role in childhood asthma	[57]
38	Patterns in the skin microbiota differ in children and teenagers between rural and urban environments	[50]
39	Seasonal Dynamics of the Airborne Bacterial Community and Selected Viruses in a Children’s Daycare Center	[65]
40	The microbial environment and its influence on asthma prevention in early life	[22]
41	Metagenomic Insights into the Bioaerosols in the Indoor and Outdoor Environments of Childcare Facilities	[66]
42	Exposure to environmental microorganisms and childhood asthma	[7]

## References

[1] Chong H.J., D’amato G., Rosário Filho N.A. (2022). Impact of the environment on the microbiome. J. Pediatr..

[2] Li S., Yang Z., Hu D., Cao L., He Q. (2021). Understanding building-occupant-microbiome interactions toward healthy built environments: A review. Front. Environ. Sci. Eng..

[3] Gilbert J.A., Blaser M.J., Caporaso J.G., Jansson J.K., Lynch S.V., Knight R. (2018). Current understanding of the human microbiome. Nat. Med..

[4] Dominguez-Bello M.G., Godoy-Vitorino F., Knight R., Blaser M.J. (2019). Role of the microbiome in human development. Gut.

[5] Robertson R.C., Manges A.R., Finlay B.B., Prendergast A.J. (2019). The human microbiome and child growth–first 1000 days and beyond. Trends Microbiol..

[6] Gaufin T., Tobin N.H., Aldrovandi G.M. (2018). The importance of the microbiome in pediatrics and pediatric infectious diseases. Curr. Opin. Pediatr..

[7] Ege M.J., Mayer M., Normand A.-C., Genuneit J., Cookson W.O., Braun-Fahrländer C., Heederik D., Piarroux R., von Mutius E. (2011). Exposure to environmental microorganisms and childhood asthma. N. Engl. J. Med..

[8] Yamazaki Y., Nakamura Y., Núñez G. (2017). Role of the microbiota in skin immunity and atopic dermatitis. Allergol. Int..

[9] Nance C.L., Deniskin R., Diaz V.C., Paul M., Anvari S., Anagnostou A. (2020). The role of the microbiome in food allergy: A review. Children.

[10] Sági V., Makra N., Csoszánszki N., Decmann A., Szabó D., Garami M. (2022). The influence of the gut Microbiome in Paediatric Cancer Origin and Treatment. Antibiotics.

[11] Mohammadkhah A.I., Simpson E.B., Patterson S.G., Ferguson J.F. (2018). Development of the gut microbiome in children, and lifetime implications for obesity and cardiometabolic disease. Children.

[12] Kelsey M.M., Zaepfel A., Bjornstad P., Nadeau K.J. (2014). Age-related consequences of childhood obesity. Gerontology.

[13] Hu M., Zhao X., Liu Y., Zhou H., You Y., Xue Z. (2023). Complex interplay of gut microbiota between obesity and asthma in children. Front. Microbiol..

[14] Ogden C.L., Carroll M.D., Fryar C.D., Flegal K.M. (2015). Prevalence of Obesity Among Adults and Youth: United States, 2011–2014.

[15] Gunnell D.J., Frankel S.J., Nanchahal K., Peters T.J., Smith G.D. (1998). Childhood obesity and adult cardiovascular mortality: A 57-y follow-up study based on the Boyd Orr cohort. Am. J. Clin. Nutr..

[16] Weihrauch-Blüher S., Schwarz P., Klusmann J.-H. (2019). Childhood obesity: Increased risk for cardiometabolic disease and cancer in adulthood. Metabolism.

[17] Liang Y., Hou D., Zhao X., Wang L., Hu Y., Liu J., Cheng H., Yang P., Shan X., Yan Y. (2015). Childhood obesity affects adult metabolic syndrome and diabetes. Endocrine.

[18] Association A.D. (2018). Economic costs of diabetes in the US in 2017. Diabetes Care.

[19] Perry R., Braileanu G., Palmer T., Stevens P. (2019). The economic burden of pediatric asthma in the United States: Literature review of current evidence. Pharmacoeconomics.

[20] Barnett S.B.L., Nurmagambetov T.A. (2011). Costs of asthma in the United States: 2002–2007. J. Allergy Clin. Immunol..

[21] Schachter A.E., Gailey A., Egendorf S.P., Mielke H.W. (2020). Mechanisms of children’s soil exposure. Curr. Probl. Pediatr. Adolesc. Health Care.

[22] von Mutius E. (2016). The microbial environment and its influence on asthma prevention in early life. J. Allergy Clin. Immunol..

[23] Robinson J.M., Barrable A. (2023). Optimising Early Childhood Educational Settings for Health Using Nature-Based Solutions: The Microbiome Aspect. Educ. Sci..

[24] Sbihi H., Boutin R.C., Cutler C., Suen M., Finlay B.B., Turvey S.E. (2019). Thinking bigger: How early-life environmental exposures shape the gut microbiome and influence the development of asthma and allergic disease. Allergy.

[25] Roof K., Oleru N. (2008). Public health: Seattle and King County’s push for the built environment. J. Environ. Health.

[26] Kaklauskas A., Gudauskas R. (2016). Intelligent decision-support systems and the Internet of Things for the smart built environment. Start-Up Creation.

[27] Kembel S.W., Meadow J.F., O’Connor T.K., Mhuireach G., Northcutt D., Kline J., Moriyama M., Brown G.Z., Bohannan B.J.M., Green J.L. (2014). Architectural Design Drives the Biogeography of Indoor Bacterial Communities. PLoS ONE.

[28] Mankiewicz Ledins P., Bhattacharya C., Dyson A., Hénaff E. (2024). Growing indoor environmental infrastructure: Designing for microbial diversity with implications for pollutant metabolism and human health. Res. Dir. Biotechnol. Des..

[29] Moher D., Shamseer L., Clarke M., Ghersi D., Liberati A., Petticrew M., Shekelle P., Stewart L.A., Group P.-P. (2015). Preferred reporting items for systematic review and meta-analysis protocols (PRISMA-P) 2015 statement. Syst. Rev..

[30] Scudellari M. (2017). Cleaning up the hygiene hypothesis. Proc. Natl. Acad. Sci. USA.

[31] Dockx Y., Täubel M., Hogervorst J., Luyten L., Peusens M., Rasking L., Sleurs H., Witters K., Plusquin M., Valkonen M. (2023). Association of indoor dust microbiota with cognitive function and behavior in preschool-aged children. Microbiome.

[32] Fu X., Du B., Meng Y., Li Y., Zhu X., Ou Z., Zhang M., Wen H., Ma’Pol A., Hashim J.H. (2023). Associations between environmental characteristics, high-resolution indoor microbiome, metabolome and allergic and non-allergic rhinitis symptoms for junior high school students. Environ. Sci. Process. Impacts.

[33] McKay J.A., Crown M., Bashton M., Pearce D., Entwistle J.A., Sangal V. (2024). Environmental microbiome in the home and daycare settings during the COVID-19 pandemic, and potential risk of non-communicable disease in children. Environ. Microbiol. Rep..

[34] Thorsen J., McCauley K., Fadrosh D., Lynch K., Barnes K.L., Bendixsen C.G., Seroogy C.M., Lynch S.V., Gern J.E. (2019). Evaluating the Effects of Farm Exposure on Infant Gut Microbiome. J. Allergy Clin. Immunol..

[35] Sobko T., Liang S., Cheng W.H., Tun H.M. (2020). Impact of outdoor nature-related activities on gut microbiota, fecal serotonin, and perceived stress in preschool children: The Play&Grow randomized controlled trial. Sci. Rep..

[36] Parajuli A., Hui N., Puhakka R., Oikarinen S., Grönroos M., Selonen V.A., Siter N., Kramna L., Roslund M.I., Vari H.K. (2020). Yard vegetation is associated with gut microbiota composition. Sci. Total Environ..

[37] Zhang M., Tang H., Chen Y., Chen Z., Xu Y., Fu X., Sun Y., Zhao Z. (2023). Impact of environmental characteristics on children’s gut microbiota—A pilot study in assessing the role of indoor microbiome and metabolites. Environ. Res..

[38] Stanaway I.B., Wallace J.C., Hong S., Wilder C.S., Green F.H., Tsai J., Knight M., Workman T., Vigoren E.M., Smith M.N. (2023). Alteration of oral microbiome composition in children living with pesticide-exposed farm workers. Int. J. Hyg. Environ. Health.

[39] Grönroos M., Parajuli A., Laitinen O.H., Roslund M.I., Vari H.K., Hyöty H., Puhakka R., Sinkkonen A. (2019). Short-term direct contact with soil and plant materials leads to an immediate increase in diversity of skin microbiota. MicrobiologyOpen.

[40] Toyoda A., Shibata Y., Matsuo Y., Terada K., Sugimoto H., Higashi K., Mori H., Ikeuchi A., Ito M., Kurokawa K. (2023). Diversity and compositional differences of the airborne microbiome in a biophilic indoor environment. Sci. Rep..

[41] Estensmo E.L.F., Morgado L., Maurice S., Martin-Sanchez P.M., Engh I.B., Mattsson J., Kauserud H., Skrede I. (2021). Spatiotemporal variation of the indoor mycobiome in daycare centers. Microbiome.

[42] Fu X., Yuan Q., Zhu X., Li Y., Meng Y., Hashim J.H., Hashim Z., Ali F., Zheng Y.W., Lai X.X. (2021). Associations between the indoor microbiome, environmental characteristics and respiratory infections in junior high school students of Johor Bahru, Malaysia. Environ. Sci. Process. Impacts.

[43] Kwam S.E. (2018). From Homes to Schools—The Impact of Ventilation and Cleaning on the Bacterial and Fungal Ecology of the Built Environment. Ph.D. Thesis.

[44] Nygaard A.B., Charnock C. (2018). Longitudinal development of the dust microbiome in a newly opened Norwegian kindergarten. Microbiome.

[45] Fu X., Norbäck D., Yuan Q., Li Y., Zhu X., Hashim J.H., Hashim Z., Ali F., Zheng Y.W., Lai X.X. (2020). Indoor microbiome, environmental characteristics and asthma among junior high school students in Johor Bahru, Malaysia. Environ. Int..

[46] Kelley S.T., Liu W., Quintana P.J.E., Hoh E., Dodder N.G., Mahabee-Gittens E.M., Padilla S., Ogden S., Frenzel S., Sisk-Hackworth L. (2021). Altered microbiomes in thirdhand smoke-exposed children and their home environments. Pediatr. Res..

[47] Sun Y., Tang H., Du S., Chen Y., Ou Z., Zhang M., Chen Z., Tang Z., Zhang D., Chen T. (2023). Indoor metabolites and chemicals outperform microbiome in classifying childhood asthma and allergic rhinitis. Eco-Environ. Health.

[48] Roslund M.I., Rantala S., Oikarinen S., Puhakka R., Hui N., Parajuli A., Laitinen O.H., Hyöty H., Rantalainen A.L., Sinkkonen A. (2019). Endocrine disruption and commensal bacteria alteration associated with gaseous and soil PAH contamination among daycare children. Environ. Int..

[49] Lehtimäki J., Laatikainen T., Karkman A., Von Hertzen L., Haahtela T., Hanski I., Ruokolainen L. (2018). Nature-oriented daycare diversifies skin microbiota in children—No robust association with allergies. Pediatr. Allergy Immunol..

[50] Lehtimäki J., Karkman A., Laatikainen T., Paalanen L., Von Hertzen L., Haahtela T., Hanski I., Ruokolainen L. (2017). Patterns in the skin microbiota differ in children and teenagers between rural and urban environments. Sci. Rep..

[51] Gardner C.M., Hoffman K., Stapleton H.M., Gunsch C.K. (2021). Exposures to Semivolatile Organic Compounds in Indoor Environments and Associations with the Gut Microbiomes of Children. Environ. Sci. Technol. Lett..

[52] Sun Y., Meng Y., Ou Z., Li Y., Zhang M., Chen Y., Zhang Z., Chen X., Mu P., Norbäck D. (2022). Indoor microbiome, air pollutants and asthma, rhinitis and eczema in preschool children—A repeated cross-sectional study. Environ. Int..

[53] Roslund M.I., Puhakka R., Nurminen N., Oikarinen S., Siter N., Grönroos M., Cinek O., Kramná L., Jumpponen A., Laitinen O.H. (2021). Long-term biodiversity intervention shapes health-associated commensal microbiota among urban day-care children. Environ. Int..

[54] Mhuireach G.Á., Fahimipour A.K., Vandegrift R., Muscarella M.E., Hickey R., Bateman A.C., Van Den Wymelenberg K.G., Bohannan B.J.M. (2022). Temporary establishment of bacteria from indoor plant leaves and soil on human skin. Environ. Microbiome.

[55] Brown M., Reeser G., Shinn L., Browning M., Schwingel A., Khan N., Holscher H. (2021). Environmental Influences on the Human Gut Microbiota: A Longitudinal Pilot Study. Curr. Dev. Nutr..

[56] Kirjavainen P.V., Karvonen A.M., Adams R.I., Täubel M., Roponen M., Tuoresmäki P., Loss G., Jayaprakash B., Depner M., Ege M.J. (2019). Farm-like indoor microbiota in non-farm homes protects children from asthma development. Nat. Med..

[57] Birzele L.T., Depner M., Ege M.J., Engel M., Kublik S., Bernau C., Loss G.J., Genuneit J., Horak E., Schloter M. (2017). Environmental and mucosal microbiota and their role in childhood asthma. Allergy.

[58] Casas L., Karvonen A.M., Kirjavainen P.V., Täubel M., Hyytiäinen H., Jayaprakash B., Lehmann I., Standl M., Pekkanen J., Heinrich J. (2019). Early life home microbiome and hyperactivity/inattention in school-age children. Sci. Rep..

[59] Roslund M.I., Puhakka R., Grönroos M., Nurminen N., Oikarinen S., Gazali A.M., Cinek O., Kramná L., Siter N., Vari H.K. (2020). Biodiversity intervention enhances immune regulation and health-associated commensal microbiota among daycare children. Sci. Adv..

[60] Beasley D., Monsur M., Hu J., Dunn R., Madden A. (2022). The bacterial community of childcare centers: Potential implications for microbial dispersal and child exposure. Environ. Microbiome.

[61] Gupta S., Hjelmsø M.H., Lehtimäki J., Li X., Mortensen M.S., Russel J., Trivedi U., Rasmussen M.A., Stokholm J., Bisgaard H. (2020). Environmental shaping of the bacterial and fungal community in infant bed dust and correlations with the airway microbiota. Microbiome.

[62] Nielsen C.C., Gascon M., Osornio-Vargas A.R., Shier C., Guttman D.S., Becker A.B., Azad M.B., Sears M.R., Lefebvre D.L., Moraes T.J. (2020). Natural environments in the urban context and gut microbiota in infants. Environ. Int..

[63] Selway C.A., Mills J.G., Weinstein P., Skelly C., Yadav S., Lowe A., Breed M.F., Weyrich L.S. (2020). Transfer of environmental microbes to the skin and respiratory tract of humans after urban green space exposure. Environ. Int..

[64] Lai P.S., Kolde R., Franzosa E.A., Gaffin J.M., Baxi S.N., Sheehan W.J., Gold D.R., Gevers D., Xavier R.J., Phipatanakul W. (2018). The classroom microbiome and asthma morbidity in children attending 3 inner-city schools. J. Allergy Clin. Immunol..

[65] Prussin A.J., Vikram A., Bibby K.J., Marr L.C. (2016). Seasonal Dynamics of the Airborne Bacterial Community and Selected Viruses in a Children’s Daycare Center. PLoS ONE.

[66] Shin S.-K., Kim J., Ha S.-M., Oh H.-S., Chun J., Sohn J., Yi H. (2015). Metagenomic Insights into the Bioaerosols in the Indoor and Outdoor Environments of Childcare Facilities. PLoS ONE.

